# Corticosteroid Treatment-Resistance in Myasthenia Gravis

**DOI:** 10.3389/fneur.2022.886625

**Published:** 2022-04-25

**Authors:** Henry J. Kaminski, Jordan Denk

**Affiliations:** Department of Neurology and Rehabilitation Medicine, George Washington University, Washington, DC, United States

**Keywords:** myasthenia gravis, corticosteroids, lymphocytes, biomarkers, clinical outcome measures

## Abstract

Chronic, high-dose, oral prednisone has been the mainstay of myasthenia gravis treatment for decades and has proven to be highly beneficial in many, toxic in some way to all, and not effective in a significant minority. No patient characteristics or biomarkers are predictive of treatment response leading to many patients suffering adverse effects with no benefit. Presently, measurements of treatment response, whether taken from clinician or patient perspective, are appreciated to be limited by lack of good correlation, which then complicates correlation to biological measures. Treatment response may be limited because disease mechanisms are not influenced by corticosteroids, limits on dosage because of adverse effects, or individual differences in corticosteroids. This review evaluates potential mechanisms that underlie lack of response to glucocorticoids in patients with myasthenia gravis.

## Introduction

Glucocorticoids (GC) are simultaneously the best and worst medications for patients with myasthenia gravis (MG). Their efficacy cannot be denied based on decades of use in clinical practice and an extensive evidence base of retrospective studies, expert opinion, and several consensus guidelines as well as a limited number of randomized trials (1–8). In 1948 ACTH was first used for MG treatment and many reports in the following two decades appreciated a therapeutic benefit (9, 10). Chronic prednisone treatment over months to years became the standard of care during the 1970's (11). Short high-dose treatment with methylprednisolone has been used (12–14). However, the usefulness of GCs is diminished by their significant adverse effects. The need to reduce corticosteroid exposure has led to the use of immunosuppressives, plasma exchange, intravenous immunoglobulin, and more recently a number of biologics for MG treatment (15, 16). The balance of effectiveness and adverse effects has led to the reduction of overall prednisone dose as a measure of efficacy in some clinical trials (17–21).

Regardless of the specific GC preparation and dosing regimen, there is a core of patients with MG who have a poor clinical response. Two large cross sectional studies of patients with MG indicated that there was a group of patients not achieving a minimal manifestation status despite higher prednisone dosage (16). Thus far, there are no patient characteristics that predict treatment-resistance (6). Shared with MG are most inflammatory or autoimmune conditions with a core of 20–30% of patients who do not improve with GC treatment (22, 23). This review will broadly assess potential mechanisms that limit treatment response to GC in MG.

## The Challenge of Defining Treatment Response

A significant challenge for MG and many disorders is the lack of reliable, objective markers of disease activity. This is in marked contrast, for example, to autoimmune thrombolytic anemia in which platelet counts track with severity of disease manifestations, respiratory parameters for asthma, or gadolinium enhancing lesions identified by magnetic resonance imaging in multiple sclerosis. Often disease severity is assessed by response to a treatment; however, this approaches a circular argument. If a drug does not work, it may simply not be targeting disease mechanisms, not accessing the site of pathology, or achieving appropriate levels to influence the disease. None of these suggest that the underlying disease mechanisms themselves are “more severe”.

Treatment resistance may stem from three broad, and potentially overlapping, reasons: (1) GC may not impact fundamental disease mechanisms, (2) excess susceptibility to corticosteroid adverse effects, which compromise ability to achieve therapeutic doses, and (3) phenotypic variations among patients that limit biological response to the GC. All these may be difficult to differentiate if severity of disease is defined as a lack of response to GC. For MG, treatment response has been assessed from various perspectives. Clinical outcome measures for MG have evolved from simple physician-centric determination of improvement to standardized strength assessment performed by trained individuals to patient reported outcomes (24, 25). Primary outcome measures for randomized trials in MG have included the total dose of GC over time, the quantitative MG Score, and the MG-Activities of Daily Living with the last of which has become the primary measure recommended by the FDA for drug approval. There has been an assumption that improvement in standardized assessments of muscle strength, as done in the QMG, would equate to improvement in patient reported outcomes, but this is not the case as appreciated by the relatively poor concordance of clinical outcome measures (26, 27). The explanation for this discrepancy lies in the complex interaction of the measurement used, disease pathology, treatment used with its adverse effects, and the individual response to disease, which includes social determinants of health and a person's personality traits. The expectation that circulating autoantibodies would be a surrogate for treatment response has not proven true. The acetylcholine receptor antibody level does not correlate with improvement (28) and the rate of change of antibody correlates only roughly (29). Small studies support muscle specific kinase (MuSK) antibodies associate with treatment response, but this has not been rigorously evaluated (30, 31). The decremental response with repetitive stimulation and abnormalities of the single fiber evaluation also do not correlate well enough with clinical disease severity to be used as a surrogate biomarker (17, 32).

## Glucocorticoid Mechanisms of Action

**Figure 1:**
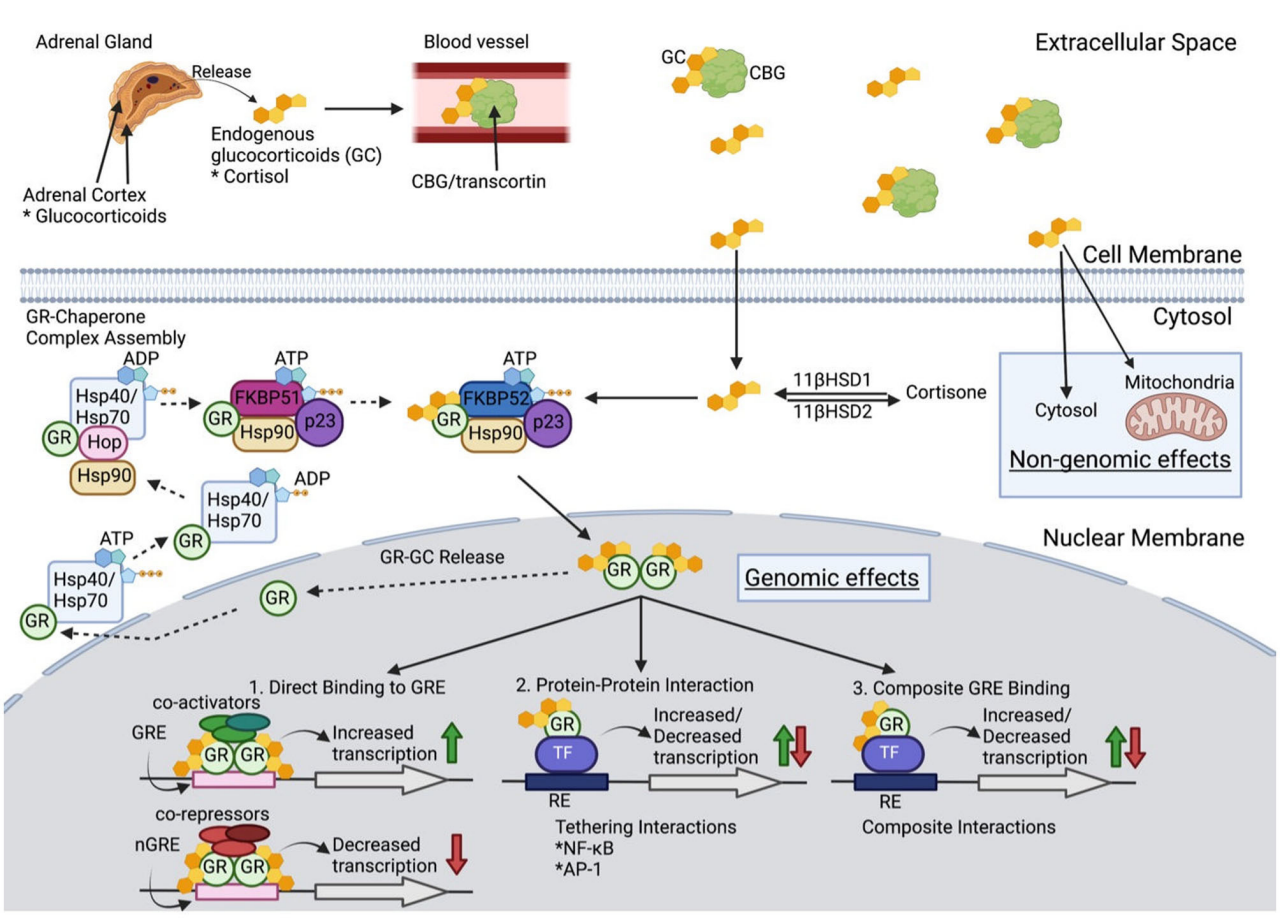
Glucocorticoid Molecular Physiology. Once released from the adrenal cortex, glucocorticoids (GC) travel through blood with the carrier protein, corticosteroid-binding globulin (CBG). Only 5% of extracellular GCs remain bioactive after binding to CBG. GC diffuse through the cell membrane to either (1) be converted into inactive cortisone *via* 11β-hydroxysteroid dehydrogenase 2, (2) have non-genomic effects in the cytosol or mitochondria, or (3) bind to the glucocorticoid receptor (GCR) as a chaperone complex to later exert genomic effects in the nucleus. When no cytoplasmic bioactive GCs are present, a multiprotein complex begins GR maturation to prepare for GC binding. Once matured, GCR's two nuclear localizations signals are exposed, which are then bound by nucleoporin and importins that translocate cytoplasmic GC into the nuclear membrane. Inside the nucleus, the GCR complex can be released, and the GR can be transported back to the cytoplasm, or the GR-GC complex can exert its function. Genomic effects include three categories: (1) direct binding to GC response elements (GREs) or negative GREs (nGREs) which recruit transcriptional co-activators and co-repressors respectively, (2) protein-protein interaction with transcription factors (TF) that modify transcription, and (3) composite interactions that involve DNA binding to GRE to alter transcription (see text for further details).

Cortisol, the endogenous glucocorticoid, is synthesized and released by the adrenal glands as regulated by the hypothalamic-pituitary-adrenal (HPA) axis (Figure 1). Corticotrophin-releasing hormone (CRH) from the hypothalamus activates corticotrophic cells of the pituitary leading to release of adrenal corticotropic hormone (ACTH), which then acts to enhance synthesis and release of cortisol from the adrenal cortex. Blood cortisol levels follow a circadian rhythm with an early morning peak and a nighttime nadir (33), and increase in response to stress including emotional reactions, physical challenges, and tissue trauma (23, 34). These diurnal fluctuations also impact the immune system and likely influence immune reactions to outside stimuli [infections) and by extension autoimmune reactions (33). The HPA axis employs a negative feedback system that occurs at both the levels of the hypothalamus and the anterior pituitary gland to moderate continued release in states of GC excess. Additionally, the hypothalamus can be stimulated by cytokine activation *via* interleukin-1 (IL-1), tumor necrosis factor (TNF), and IL-6 (35) as would occur in inflammatory and autoimmune diseases. Psychological stress also increases GC production due to increased noradrenaline levels, which further stimulate CRH and cause an increase in pro-inflammatory cytokines, all of which stimulate the HPA axis (33).

Cortisol binds the carrier protein, corticosteroid-binding globulin (CBG), for its distribution *via* the circulation. Bound cortisol is inactive, and only the small fraction of unbound GC, which is lipophilic, diffuses readily across cell membranes. Cytoplasmic cortisol binds to the GC receptor (GCR). The bound GC and GCR impact biological processes through (1) activity as a transcription factor binding to GC response elements of numerous genes, (2) interactions with other transcription factors including nuclear factor-κB (NF-κB) and activator protein 1 (AP-1), and (3) repression of gene transcription through binding of inhibitory GC response elements and binding of other transcription factors to prevent their action (35). GCs have been estimated to impact expression of 20% of the genome (36). GCs also act through non-genomic mechanisms. The lipophilic properties of GCs lead to their ability, in the absence of the glucocorticoid receptor (GR), to enter lipid membrane, which alters membrane fluidity and interaction with membrane bound proteins, including ion channels. The alteration of sodium and calcium transfer appears to be a factor in mediating some anti-inflammatory effects. To add to the complexity of GC influences each cell differs in the nature of transcriptional factors and other proteins for the GC to interact. Given their numerous tissue targets, excess glucocorticoid states, whether endogenous as in Cushing's syndrome or exogenous provided as prednisone, can lead to numerous adverse effects with wide inter-individual variation for treatment response. Synthetic GC, i.e., prednisone and dexamethasone, are not subject to endogenous inhibitors of cortisol activity making them more potent anti-inflammatory agents. Prednisone binds the GCR with higher affinity and mineralocorticoid receptors with lower affinity than does cortisol, thereby limiting mineralocorticoid-based complications.

The GCR is key in mediating many of the actions of GC. The protein has three functional regions. (1) The constitutively active ligand-independent activation domain (AF-1) is located in the N-terminal region and is bound by the transcriptional machinery and coregulators. (2) The DNA-binding domain allows for binding of the GR to DNA and regulatory proteins. (3) Ligand-binding domain of the C-terminus also serves to interact with other transcriptional proteins, chaperone proteins, and coregulators. The GR protein activity is subject to regulation by phosphorylation, ubiquitination, and acetylation. The human GR gene transcript undergoes alternative splicing to generate GRα and GRβ isoforms, each with specific activity. The isoforms are nearly identical through amino acid 727, but GRα contains an additional 50 amino acids, and GRβ differs with an additional 15 non-homologous amino acids. GRβ is present in the nucleus and is transcriptionally active with the capability to repress or activate genes regulated by GRα. GRβ can inhibit GRα activity. Proinflammatory cytokines and other signals increase the expression of GRβ and mediate GC resistance (37). Other GCR isoforms exist but are less well understood and have not been associated with GC resistance. There are an increasing number of proteins being identified, which bind the GCR and its complex with GC and are likely to influence GC activity. A detailed discussion of these is beyond the scope of this review and reader should see the excellent summary of Petta et al. (35).

The GCR suppresses pro-inflammatory pathways supported by NF-κB, AP-1, and MAPK (23). Each of these major pathways influence cell survival, apoptosis, proliferation, differentiation and production of activate cytokines, chemokines, and other key aspects of inflammation. Although all have predominant pro-inflammatory, a chronic high level of NF-κB activity may lead not only to chronic inflammation, but also to GC resistance by blocking the GCR signaling pathway. Such chronic low level inflammation has been implicated in the pathogenesis of many diseases (38). GC also has *pro-inflammatory* effects in certain situations including the dose of GC and timing during the development of inflammation (39). For example, low dose GC will enhance delayed-hypersensitivity in rat models, but their chronic, high dose administration will enhance the response (40).

GC have significant influences on cellular immunity. GC inhibit dendritic cell maturation through reduction of expression of MHC class II and costimulatory molecules. They also have complicated effects on T cells, which include interference in TCR signaling leading to reduced T cell activity, but GC appears to have a suppressive effect on Th1 and Th17 cells, but promote Th2 and Treg cells. GC treatment increases frequencies of circulating Treg cells, which is likely GC mediated increase in forkhead box P3 (FOXP3) through upregulation of GILZ87 (23). Thymocytes are particularly sensitive to GC-mediated apoptosis. The details of GC effects on B cells is being elucidated. GC treatment reduces antibody concentrations in circulation and immature B cells, which express GCR, are particularly sensitive to induced apoptosis in contrast to more mature B cells and plasm cells. However, emerging literature supports that GC can have pro-inflammatory effects. GC enhances sensitivity of some cytokine receptors, while reducing circulating levels of these cytokines. Expression profiling indicates that gene expression of innate immunity including complement components, receptors of chemokines and cytokines, are upregulated, while T cell pathway genes are increased. Cain and Cidlowski propose that in the normal condition immune cells are sensitized to detected infections and other harmful signals leading to tissue damage and thereby the immune system can react rapidly (23). In a pro-inflammatory state, stress-induced increases in cortisol or exogenous GC will reduce the acute immune response. This dual state of pro- and anti-inflammatory effects leads to the complicated effects of exogenous GC treatments in autoimmune diseases and the impact of GC dosage and duration of treatment.

## Steroid-Resistance in Myasthenia Gravis

A detailed analysis of MG pathophysiology is beyond the scope of this discussion but are reviewed in the context of treatment resistance. The authors recommend readers see a recent review by Huijbers et al. (41). As mentioned above, there are three categories of explanation why patients with MG would not respond to GC treatment. The disease-causing mechanisms are not influenced by GC, the adverse effects of GC are not tolerated leading to an inadequate dose, or there are individual traits which limit the effect of GC treatment.

### Underlying Pathology Does Not Respond to Corticosteroids

Among the best examples of apparently similar inflammatory diseases with contrasting responses to GC treatment are inflammatory pulmonary conditions, which account for about 60% of prescriptions for oral GC in the United Kingdom (42). Asthma, chronic obstructive pulmonary disease, interstitial pulmonary fibrosis, and cystic fibrosis demonstrate inflammatory infiltrates (43) in the lung with an expectation that GC therapy would moderate the severity of each disease, but a significant benefit is only appreciated in patients with asthma.

As an autoimmune disease with a preponderance of patients improving with GC treatment, there appears to be no a priori reason for GC to be unable to target the immunopathology of MG. However, the possibility that some mechanisms driving pathology, which are not amenable to GC treatment should not be discounted. MG is not a single disease, but rather has subgroups defined by age, thymic pathology and autoantibody status. Existing data supports MuSK MG being primarily a disease of short-lived plasma cells, which are more sensitive to GC treatment, compared to long-lived plasma cells of AChR antibody positive MG (31, 44). The better response to anti-CD20 treatment of MuSK MG than AChR MG supports that short-lived CD20 expressing plasma cells are critical in disease pathology compared to long-lived plasma cells, which do not express CD20 (45, 46). GC-resistance may change over time with the potential for long-lived plasma cells becoming the major driver of pathology, compared to earlier in the disease may also induce resistance itself. Other than plasma cell lineage factors disease factors, which are not amenable to GC sensitivity are not known.

### Adverse Effect Susceptibility

One aspect of GC resistance, which should not be overlooked, is the variation in susceptibility to adverse effects, which then compromises ability to achieve therapeutic doses. Despite the well-appreciated adverse effects of GC treatment, there is limited data on the inter-individual susceptibility to adverse effects. Upwards of one to two thirds of patients with MG hav e adverse effects related to GC therapy (19, 47). The major risk factor for GC morbidity is the cumulative dose of GC, but even with lower dose regimens of 20–30 mg of prednisone vs. the historical standards 60–80 mg per day dosing, intolerable adverse effects occur (16, 48, 49). The most common adverse effects are weight gain, Cushingoid appearance, and skin changes including acne, while more medically severe effects, but rare complications, include gastric and esophageal irritation, compression fractures, and aseptic necrosis of the femoral head. Between these ends of severity are worsening hypertension, diabetes, glaucoma and cataract formation. Poorly-documented adverse effects, which occur in essentially all patients, are insomnia and mood changes from irritability and various degrees of depression. A study of over a thousand rheumatoid arthritis patients found a dose-dependent relation with Cushingoid features, peripheral edema, skin bruising and threshold effect of 7.5 mg per day with glaucoma, depression and hypertension, while even five mg per day increased incidence of weight gain and even a lower dose of cataract formation (50). McDowell et al. evaluated adverse effects in a population of patients with severe asthma and using a quantitative instrument specific for GC treatment complications confirmed significant inter-individual variability in adverse effects, which is consistent with the long-standing clinical impression. The inter-individual susceptibility to adverse effects and treatment resistance are intertwined from the clinician and patient perspective but biological mechanisms that drive improvement vs. complications are likely distinct. GC differentially influence gene expression of pathways, which moderate inflammatory and adverse effects (51) with adverse effects primarily associated with the transactivation of genes by the corticosteroid, which has led to attempts to engineer compounds that support suppression of pro-inflammatory gene transcription, but limit transactivation (52–54).

### Sensitivity to Treatment Effect of Glucocorticoids

As should be clear from the summary of GC action, there is the potential for GC efficacy to be compromised at many steps from administration to final effector mechanisms. Below we review the presently known mechanisms of GC resistance that may impact efficacy for MG.

#### GC Metabolism

Despite decades of use, there is relatively poor characterization of the impact of GC metabolism on therapeutic benefit. Efficacy properties of any drug begin with its pharmacokinetic profile. Exogenous GC are not subject to endogenous moderators of cortisol (55). Prednisone and prednisolone are the most frequently used GC in treatment of MG with both drugs rapidly absorbed after oral ingestion. Prednisone is converted to prednisolone rapidly by the action of 11β-hydroxysteroid dehydrogenase with a peak blood concentration within 3 h. High inter-individual difference in bioavailability of prednisone has been documented (56). Prednisone is cleared primarily by hepatic metabolism by the P450 system and drugs, which block or enhance P450 enzymes will modify the half-life of the drug. Prednisone itself may modify xenobiotic pathways that metabolize the drug, which further enhances the complexity of inter-individual variation of efficacy (57). In addition, both the GCR and xenobiotic receptor activation inhibit the activity of NF-κB, a master regulator of the immune response (58). Also, NF-κB activation reciprocally inhibits xenobiotic metabolism, creating a complex feedback loop. The simple variation of metabolism of prednisone could impact its efficacy in individual patients with MG. Genetic differences in drug metabolism are being appreciated but have not yet reached an understanding to guide GC therapy.

#### Pharmacogenetics and Glucocorticoid Resistance

Genetic variations are well-appreciated to influence drug responses or adverse effects to GC but have yet to be defined well enough to guide practice. Polymorphisms in the GCR gene are associated with response to GC in ulcerative colitis and rheumatoid arthritis (59–61), and we also found this to be the case in GC treatment response in patients with MG (62) (Table 1). The only other gene with genetic polymorphisms associated with treatment response in MG is osteopontin (63). Circulating GRβ levels have been found to be associated with GC resistance in rheumatoid arthritis, SLE, and asthma (64–66). Hypomethylation of NLRP3 gene promoter discriminates glucocorticoid-resistant from GC-sensitive idiopathic nephrotic syndrome patients (67). P53 interacts with GR to promote anti-inflammatory pathways and patients with rheumatoid arthritis who did not respond to GC treatment showed reduced p53 expression levels in blood mononuclear cells (68). Genetic variations, including ones that vary in significance based on sex, are increasingly being appreciated in response to GC therapy but have yet to guide treatment decisions. The response to GC therapy may wane over time appreciated for some conditions is produced by a downregulation of the GRα (69).

**Table 1:** Examples of genes with single nucleotide polymorphisms associated with GC resistance.

**Gene**	**Protein**	**Disease GC Resistance**
*NR3C1*	Glucocorticoid Receptor	MG, pediatric nephrotic syndrome
*FKBP5*	FK506 binding protein 5	Inflammatory bowel disease
*IL-4*	Interleukin-4	Nephrotic syndrome
*IL-6*	Interleukin-6	Nephrotic syndrome
*MIF*	macrophage migration inhibitory factor	inflammatory bowel disease, rheumatoid arthritis
*GLCCI1*	Glucocorticoid Induced 1	Asthma
*MDR1*	P-glycoprotein	Nephrotic syndrome, inflammatory bowel disease, rheumatoid arthritis
*NR1I2*	Pregnane X receptor	Nephrotic syndrome

#### Lymphocyte Sensitivity

Investigations of cultured lymphocytes of patients with rheumatoid arthritis, inflammatory bowel diseases and systemic lupus demonstrate a sensitivity to *in vitro* lysis when cultured with GC, which correlates with the clinical benefit observed in these patients (42, 70, 71). Of note, the *in vitro* sensitivity is observed in non-disease control subjects and therefore is not a function of disease activity. Studies of African Americans with asthma show less *in vitro* sensitivity to GC, which again correlates with poorer clinical response to GC therapy (72). Glycosphingolipid metabolism, urea cycle, and pentose phosphate pathways are associated with *in vitro* glucocorticoid resistance in pregnant African American women (73). Differences in transcription of NF-κB and other genes are associated with the degree of lymphocyte sensitivity to glucocorticoids (70, 74).

#### Sex and Gender Differences in Autoimmunity and Glucocorticoid Resistance

Sex refers to characteristics specific to biologically determined properties of the sex chromosomes. Gender encompasses biological differences coupled with social and cultural factors, which define women and men. Under the age of 40 years about two thirds of patients with MG are women while with advancing age the gender discrepancy begins to shift toward men. Rheumatoid arthritis and multiple sclerosis share a similar distribution, in contrast, women account for over 90% of cases of SLE and Sjogren's. These observations support that there are fundamental gender differences in susceptibility to initiation and maintenance of autoimmune disorders that are dependent on the specific disease. There is an ever-increasing appreciation of the differences in the immune responses of females and males, which span species from Drosophila to humans. Females develop more intense innate and adaptive immune reactions than males allowing for better clearance of infectious agents as well as greater responses to vaccinations; however, this comes at the price of greater susceptibility to autoimmune process (75, 76). Sex hormones and immune system related genes on the X chromosome hosts are factors, which drive these differences. The impact of sex hormones on autoimmunity is illustrated by the general observation that disease severity is reduced during pregnancy and exacerbate post-partum. Pregnancy also leads to the transmission of fetal cells to the mother and these foreign cells can persist for decades. Maternal cells also persist in individuals at very low levels throughout postnatal development. The maternal receipt of fetal cells likely expands immune tolerance in the mother during pregnancy, but they may also contribute to increased risk of autoimmune disease in women of child bearing years (77). Epigenetic factors impact gene expression on the X chromosome and thereby provide mechanisms on how the environment may shape gender differences in autoimmunity (78–80).

The severity of autoimmune diseases vary based on gender. Men with psoriasis, multiple sclerosis and SLE have a worse prognosis, in contrast to there not being a difference in rheumatoid arthritis (79). Young women also have a poorer response compared to men to GC therapy for inflammatory bowel disease (81). Mortality rates generally are higher among women with autoimmune diseases, but this data is difficult to interpret as to whether biological, social, comorbidies, or other factors drive these observations. A patient reported registry study indicated that women with MG have a poorer quality of life (82), but there is limited data as to whether women respond less well to treatment. Women report a poorer response to overall treatments for MG and have greater adverse effects from prednisone (82, 83). Endogenous and exogenous GC influence gene expression, including those of the immune system, in a sex specific manner (84).

## Clinical Consequences

Identifying treatment-resistant patients prior to initiation of GC is presently not possible and therefore, the clinician needs to be proactive in discontinuation of prednisone treatment to prevent greater adverse effects than can be balanced by benefit. Consensus guidelines recommend moving to alternative therapies when initial GC therapy at “adequate” dosing does not improve or worsens the patient's condition or if adverse effects are deemed intolerable by patient or physician (8). The consensus guideline provides options of slow, alternate dose escalation or a high-dose rapid induction. No specific time-frame for improvement, level of response, or severity of adverse effects is defined. A responsive patient to prednisone usually does so in 4–6 weeks after prednisone initiation. This may not be complete, but physician and patient should expect a situation close to minimal manifestations. Again, both patient and clinician should guard against being content with significant improvement from a poor baseline and accepting disability.

The MG community is blessed with therapeutic options for GC treatment resistance, which are detailed in a number of recent reviews (15, 21). For all AChR-Ab positive patients under 65 years of age as was defined in the MGTX study this would mean a thymectomy regardless of response to prednisone (19). Relatively, rapidly acting approaches as intravenous immunoglobulin, plasma exchange, complement inhibition or FcRn blockers, should be used for patients presenting with significant disability and an initial poor response to prednisone. However, none of these treatments will lead to remission and therefore, for long-term reduction of antibody producing cells, immunosuppressives and B cell ablation therapy should be considered. Tapering of prednisone should begin with initiation of additional therapies and its speed dependent on the nature of the additional treatment and the expected onset of action.

## Concluding Remarks

MG therapeutic development is making incredible advances (21) with agents that specifically target effector mechanisms as well as autoantibody producing cells and attempts to reestablish tolerance. Despite the new drugs approved, and ones on the horizon, GC treatment will continue to be the primary therapy used for MG care for the foreseeable future (8). Detailed investigation of patients who demonstrate differential responses to GC offer a powerful set of experiments to understand MG mechanisms and further define differences, which will allow development of personalized medicine for patients. The application of broad spectrum proteomic, genomic, metabolomic, and microbiome approaches linked to precise clinical characterization will be key to elucidating subtle differences in disease mechanisms and treatment response.

## Author Contributions

HK formulated review, wrote the majority of the manuscript, and reviewed the entire final manuscript. JD drafted portions of the text, developed the figure and legend, and reviewed the final submission. Both authors contributed to the article and approved the submitted version.

## Funding

This work was supported by the MGNet a member of the Rare Disease Clinical Research Network Consortium (RDCRN) NIH U54 NS115054. Funding support for the DMCC was provided by the National Center for Advancing Translational Sciences (NCATS) and the National Institute of Neurological Disorders and Stroke (NINDS).

## Conflict of Interest

HK is a consultant for Roche, Cabeletta Bio, Lincoln Therapeutics, Takeda and UCB Pharmaceuticals; and is CEO and CMO of ARC Biotechnology, LLC based on US Patent 8,961,98. He is principal investigator of the Rare Disease Network for Myasthenia Gravis (MGNet) National Institute of Neurological Disorders and Stroke, U54 NS115054 and Targeted Therapy for Myasthenia Gravis. R41 NS110331-01 to ARC Biotechnology. The remaining author declares that the research was conducted in the absence of any commercial or financial relationships that could be construed as a potential conflict of interest.

## Publisher's Note

All claims expressed in this article are solely those of the authors and do not necessarily represent those of their affiliated organizations, or those of the publisher, the editors and the reviewers. Any product that may be evaluated in this article, or claim that may be made by its manufacturer, is not guaranteed or endorsed by the publisher.
